# Effect of plant diversity on the diversity of soil organic compounds

**DOI:** 10.1371/journal.pone.0170494

**Published:** 2017-02-06

**Authors:** Lamiae El Moujahid, Xavier Le Roux, Serge Michalet, Florian Bellvert, Alexandra Weigelt, Franck Poly

**Affiliations:** 1 Université de Lyon, Université Lyon 1, CNRS, INRA, Laboratoire d’Ecologie microbienne, UMR 5557 CNRS, UMR 1418 INRA, Villeurbanne, France; 2 Université de Lyon, Université Lyon 1, UMR5557 CNRS, UMR 1418 INRA, Centre d’Etude des Substances Naturelles, Villeurbanne, France; 3 Department of Systematic Botany and Functional Biodiversity, Institute of Biology, University of Leipzig, Leipzig, Germany; 4 German Centre for Integrative Biodiversity Research (iDiv) Halle-Jena-Leipzig, Leipzig, Germany; Institute of Tibetan Plateau Research Chinese Academy of Sciences, CHINA

## Abstract

The effect of plant diversity on aboveground organisms and processes was largely studied but there is still a lack of knowledge regarding the link between plant diversity and soil characteristics. Here, we analyzed the effect of plant identity and diversity on the diversity of extractible soil organic compounds (ESOC) using 87 experimental grassland plots with different levels of plant diversity and based on a pool of over 50 plant species. Two pools of low molecular weight organic compounds, LMW1 and LMW2, were characterized by GC-MS and HPLC-DAD, respectively. These pools include specific organic acids, fatty acids and phenolics, with more organic acids in LMW1 and more phenolics in LMW2. Plant effect on the diversity of LMW1 and LMW2 compounds was strong and weak, respectively. LMW1 richness observed for bare soil was lower than that observed for all planted soils; and the richness of these soil compounds increased twofold when dominant plant species richness increased from 1 to 6. Comparing the richness of LMW1 compounds observed for a range of plant mixtures and for plant monocultures of species present in these mixtures, we showed that plant species richness increases the richness of these ESOC mainly through complementarity effects among plant species associated with contrasted spectra of soil compounds. This could explain previously reported effects of plant diversity on the diversity of soil heterotrophic microorganisms.

## Introduction

During the past decades, many studies have analysed the role of biodiversity on ecosystems, demonstrating that a loss in biological diversity can affect ecosystem characteristics and functioning [1–3]. Key mechanisms underlying the effects of plant diversity on ecosystems have been debated. In particular, several authors distinguished causal mechanisms such as facilitation or complementarity involving interactions between several species which potentially results in overyielding, i.e. better performance for assemblages of species than the best one observed for monoculture of each of the species [4]; and probabilistic mechanisms through a sampling or selection effect, in which the probability of finding a species with high performance is higher in more diverse communities, the presence of such species explaining the high mean level of functioning of diverse mixtures. Williams and McCarthy [5] gave a general definition of complementarity as “a property of set of objects that exists when at least some of the objects in one set differ from the objects in another set”. In many ecological studies, these 'objects' refer to plant species and 'property' to the level of a process like primary productivity, complementarity being assessed by comparing the performance of monocultures as compared to mixtures [6–7]. Plant diversity effects and underlying mechanisms have also been studied focusing on other ecosystem functions including nutrient retention and partitioning [8], soil microbial activities [9], and emissions of chemical compounds [10–11], these effects having great ecological importance [12].

In this context, the effects of plant diversity on some key soil characteristics and functions such as nitrogen retention [13], carbon (C) sequestration [14] or soil biota diversity [15] have been increasingly studied. In contrast, the effect of plant diversity on the belowground resource availability and particularly the fine characteristics of soil organic matter has been overlooked, and studies on the effects of environmental gradients on soil biodiversity generally disregard the importance of changes in plant diversity on these fine characteristics of soil organic matter [16]. However, by supplying leaf and root litter and rhizodeposits to the soil, plants represent the major entering pathway of organic matter into the soil. Soil organic matter, SOM, is a mixture of a variety of compounds that could be classified into two types: humic and non-humic substances. Non-humic organic molecules are released from cells or fresh residues and represent an active or easily decomposed fraction of SOM [17]. A considerable spectrum of low-molecular weight (LMW) compounds such as simple organic acids, phenolic compounds, amino acids, carbohydrates and lipids hence constitutes the active part of SOM in the soil solution [18] which is the most dynamic component of soil C [19]. These compounds critically contribute to the cycling of C, N and P and represent energy resources easily available for soil heterotrophic organisms for which growth is often limited by resource availability [20]. Because the nature of LMW compounds released to soil via plant litter or root exudation depends on plant species identity [21], plant diversity could strongly influence the composition and diversity of soil non-humic organic molecules. This could have major consequences for the functioning and biodiversity of soil and ultimately the ecosystem, because the diversity of LMW organic compounds strongly influences the functioning [22–23] and the diversity of soil organisms [24]. However, no comprehensive study has assessed the effect of plant diversity on the diversity of extractible soil organic compounds so far.

Given the differences in litter and exudates quality already reported among plant species, we hypothesized that an increase of plant species richness would induce an increase of the richness of soil LMW organic compounds. This hypothesis is implicit in many published studies [25–26] but to our knowledge never demonstrated. It is based on the presumption that a more diverse plant community brings a high number of organic compounds into soil both through a complementarity effect [27], i.e. different spectra of organic compounds brought into soil by different plant species, and a selection effect [27], i.e. diverse plant communities would more often include at least one plant species producing a high number of compounds. In this context, our objectives were (1) to assess the effect of plant diversity on the diversity of extractible soil organic compounds of low molecular weight, LMW, and (2) to identify the key mechanisms explaining the effect of plant species on the diversity of these compounds. We assessed the diversity of soil organic compounds using two different chromatographic techniques, GC-MS and HPLC-DAD, allowing to study two pools of low molecular weight organic compounds, LMW1 and LMW2 respectively, that include organic acids, fatty acids and phenolics (with more organic acids and more phenolics in LMW1 and LMW2 respectively). These techniques allow the accounting for at least i) phenolic compounds which represent an important portion of the C pool entering into the soil [28] and ii) soil organic acids derived mainly, although not exclusively, from plants (root exudation, plant cell lyses…) and which are of paramount importance for the growth and developments of many soil organisms [29–31]. We assessed the diversity of these soil compounds for a total of 87 plots of experimental grassland plant communities corresponding to a range of sown plant species richness from 0 to 16 and based on a pool of over 50 grassland plant species (Jena biodiversity experiment; [32]).

## Materials and methods

### Ethics statement

No specific permits were required for the described field studies. The Jena field site is a former arable land owned by an agricultural collective. The ground is leased by the Research consortium of the Jena experiment for scientific purpose including soil sampling and other experimental manipulations. The site is not protected in any way. The areas studied do not involve any species endangered or protected in Germany.

### Experimental site

The study was carried out as part of a large biodiversity experiment conducted in Jena, Germany (50°57’N, 11°37’W, elevation 130 m) for investigating the role of plant diversity for grassland ecosystem functioning [32]. The mean annual air temperature is 9.3°C (1961–1990) and the mean annual precipitation is 590 mm. The soil of the field site, located on the floodplain of the Saale River at the northern edge of Jena (altitude 130 m NN, Thuringia, Germany), is an Eutric Fluvisol (FAO-Unesco classification) developed from up to 2 m thick loamy fluvial sediments. The site has been used as an arable field for the last 40 years and converted from grassland in the early 1960ies [32]. The experimental study system was derived from a typical Central European mesophilic grassland community as it was traditionally used for haymaking. Sixty common plant species of this community were selected (S1 Table) and divided by ordination using 17 species traits into four functional groups which could be identified *a posteriori* as grasses, legumes, tall non-leguminous herbs, and small non-leguminous herbs [32].

The Jena Experiment was sown in 2002. The experimental area was partitioned into four blocks, containing large (20 m x 20 m) or small (3.5 m x 3.5 m) plots of experimental plant communities of 0 (bare soil), 1, 2, 4, 8 and 16 species. In all mixtures, plant species were sown at maximum evenness. All plots were weeded regularly, thus maintaining plant species richness at the planned levels or below in cases where plant species did not establish or go extinct. The experimental plant communities were mown twice per year and were not fertilized.

### Soil sampling

For the present study, 4, 16, 16 and 14 large plots with respectively 0, 1, 2 and 16 plant species were sampled at the date of experimental set up (= 50 large plots). In addition, we sampled 37 small plots cultivated with the monocultures missing in large plots (= total of 87 plots). We assessed the realized (actual) plant species richness in 2006 from data of species-specific biomass [33]. Plant species whose biomass was <5% of the total plant biomass in the plot were excluded for the computation of actual plant richness since such species hardly influenced the characteristics of composite soil samples taken at plot scale. We tested other threshold values (3 to 10%) to compute actual richness and found that the value used here did not affect our conclusions.

Soil samples (0–8 cm depth) were collected using corers (8 cm diameter) in October 2006. Ten samples were taken along a transect of 9 m for each large plot (total of 500 soil cores), and five samples were taken each 50 cm for each small plot (additional 210 soil cores). For each plot, a composite soil core was obtained by pooling one half of each core taken in the plot. For each composite, fresh soil was sieved using 2-mm mesh size, homogenized and stored at -20°C before extraction of soil organic compounds.

### Extraction of organic compounds from soil

For each composite sample, 10 g (equivalent dry soil) were transferred into stainless steel extraction cell. Soil organic matter and soil soluble organic compounds were extracted by accelerated solvent extraction (Dionex ASE 200^™^) using dichloromethane and a mixture of methanol and water (1:3 v/v), respectively. Extractions were performed sequentially at 50°C and 1500 psi with a 5 min heating phase followed by 10 min static extraction, with first 5ml of the methanol-water mixture. The cell was then rinsed with 17 ml of the same solvents and the soil solution containing the extracted compounds was flushed from the extraction cell into a collection vial using N_2_ at 1500 psi. The sample was extracted again using fresh solvent and flushed into a second collection vial. A second sequential extraction was then achieved on the same soil sample cell using dichloromethane and with the same procedure, the extracted compounds being flushed into a third and a forth collection vials.

### Preparation of soil extracts and chromatographic analysis

Aqueous-methanolic extracts (polar extracts) were filtered using a Millipore filter (Polytetrafluoroethylene, PTFE, membrane; 40 μm). Then each solution was dried at 40°C in a centrifugal vacuum concentrator (Speed-Vac^®^) to evaporate first the methanol; the aqueous part remaining was freeze-dried (Martin Christ Alpha 1–4 LSC). Dichloromethane extracts (apolar extracts) were dried at 40°C in the centrifugal vacuum concentrator; dried extracts were then dissolved in 5 ml methanol before being filtered (PTFE membrane; 40 μm). These solutions were finally dried in a centrifugal vacuum concentrator. After weighing the dry extracts, polar and apolar extracts were dissolved in 2 ml of the *ad hoc* solvents and collection vials were pooled to obtain 2 extracts for each soil sample: a polar extract and an apolar extract. These extracts were dried and concentrated at 4 mg/ml in methanol.

Compounds analysis was first performed by Gas Chromatography—Mass Spectrometry (GC-MS) using a gas chromatograph HP 6890 coupled with a HP 5973 mass spectrometer (Agilent Technology) controlled by the HP Chemstation software. GC runs were performed on a DB-FFAP column (30 m, 250 μm internal diameter, 0.25 μm film thickness; Agilent Technology). The injection was operated in splitless mode at 60°C with a constant helium carrier gas flow adjusted to 1.0 mL/min. Oven temperature was programmed to increase from 60°C to 260°C at 10°C/min. The injected volume was 1 μl. For mass spectrometry we used Electron ionization mode at 70eV and the scan range was 30–600 amu. Chromatograms obtained were integrated after standardization using the HP Chemstation software.

In addition, UV absorbing compounds were analyzed by High Performance Liquid Chromatography (HPLC) using an Agilent 1200 series HPLC equipped with a diode array detector (DAD) (Agilent technologies). The analysis was carried out at room temperature using a Nucleodur Sphinx C18 column (250 x 4.6 mm; 5μm; Macherey-Nagel). For polar and apolar extracts of each sample, 20 μl were injected and the column was eluted by two optimized procedures depending on the extract’s type (see Appendix 1). UV data were recorded between 200 to 600 nm. Chromatograms, processed at 280 nm, were integrated after standardization using the Chemstation software. The low molecular weight compounds detected using GC-MS are referred to hereafter as LMW1 organic compounds and those detected using HPLC-DAD are referred to as LMW2 compounds. These pools of compounds include organic acids, fatty acids and phenolics, with more organic acids and more phenolics in LMW1 and LMW2, respectively. Note that the methods used do not allow characterization of amino acids.

### Data analysis

For polar and apolar extracts, the chromatograms of the different soil samples were compared and the retention time of each peak was aligned to construct an organic compounds matrix.

Block effect on soil compound diversity was first tested by analysis of variance, which was not significant (p = 0.29 and p = 0.32 respectively for soil LMW1 and LMW2 compound richnesses). Similarly, block effect on soil compound composition was tested by analysis of similarity (ANOSIM) using the Bray-Curtis coefficient (PRIMER-E software, Plymouth, UK), which was not significant for the composition of the LMW1 compounds (p = 0.12) but was significant for the LMW2 compounds (p = 0.001). For analyzing plant diversity effect on organic compound diversity, only compounds not detected in bare soil extracts were considered.

The mean richness of both types of soil compounds was compared between plant functional groups or between plant families using analysis of variance followed by Tukey’s test. Data normality was tested using the Shapiro test. The organic compound compositions between plant functional groups or between plant families were compared using ANOSIM.

We then tested the effect of plant diversity on the richness of soil LMW1 and LMW2 compounds using linear or logarithmic regression. Given the observed relationships, we tested to what extent soil compound richness for a given plant assemblage could be explained by a complementarity effect among plants present in the assemblage as presented in S1 Fig. In this case, it was assumed that each plant species is associated with a given soil compound profile and that a mixture of plant species is associated with the corresponding mixture of compounds brought in by each plant species of the assemblage. For a given plot, only plant species with biomass representing more than 5% of total plant biomass were considered for the analysis.

## Results

### Effect of plant species identity, functional group and family on soil organic compound richness in monocultures

**Fig 1:**
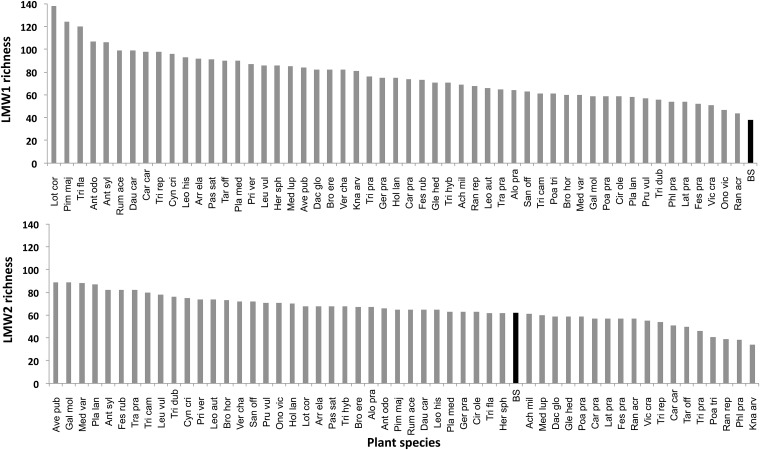
Richness of soil LMW1 compounds (upper panel), and LMW2 compounds (lower panel), observed for a range of plant species grown as monocultures. BS refers to bare soil (i.e. non planted since the beginning of the experiment; black bars). The abbreviations of the plant names are explained in Appendix 1.

LMW1 compound richness was higher for each of the 53 different plant species studied as monocultures than for bare soil: LMW1 richness ranged from 38 compounds for bare soil up to 138 compounds for *Lotus corniculatus* (Fig 1a). LMW2 richness showed less variability between plant species, varying between 34 and 89 (Fig 1b). Moreover, the number of LMW2 compounds detected for bare soil was 51, which was higher than values reported for 17% of plant monocultures.

**Fig 2:**
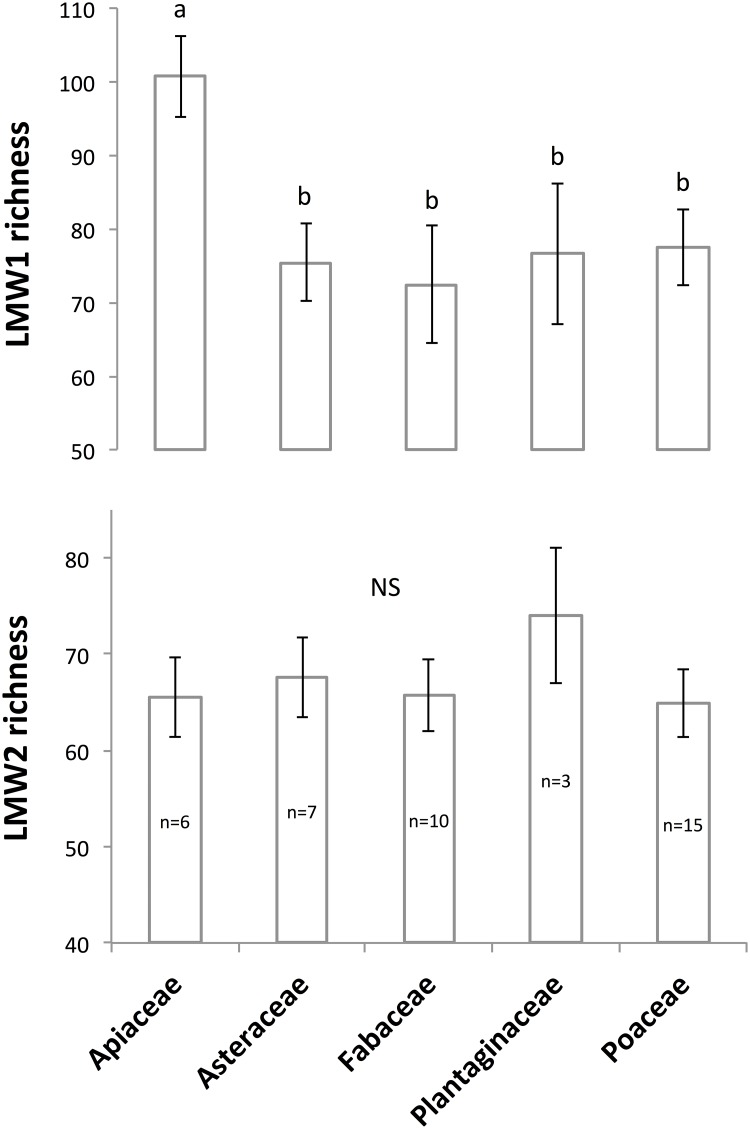
Effect of the botanical family on the richness of soil LMW1 compounds (Left) and soil LMW2 compounds (Right) according to the results obtained for plant monocultures. The number of replicates, i.e. different plant species per family, is indicated into boxplots. Different letters indicate significant differences at p = 0.06. NS: not significant (p = 0.57, 0.26, and 0.996 for panels b, c and d respectively).

LMW1 compound richness marginally differed (p = 0.069) between plant families (Fig 2). In particular, a significantly higher LMW1 richness was observed for plant species belonging to the *Apiaceae* compared to other families (Fig 2a). In contrast, LMW1 richness did not differ between the 4 plant functional groups defined during the set up of the Jena biodiversity experiment (p = 0.26). Plant family and plant functional group did not influence the richness of LMW2 (p = 0.57 and p = 0.99, respectively).

The composition of soil LMW1 compounds marginally differed between plant families (p = 0.09) and did not differ between plant functional groups (p = 0.115) (not shown). The composition of LMW2 compounds was not influenced by plant functional group or plant family (p = 0.19 and p = 0.40 respectively).

### Effect of plant diversity on the diversity of soil organic compounds

**Fig 3:**
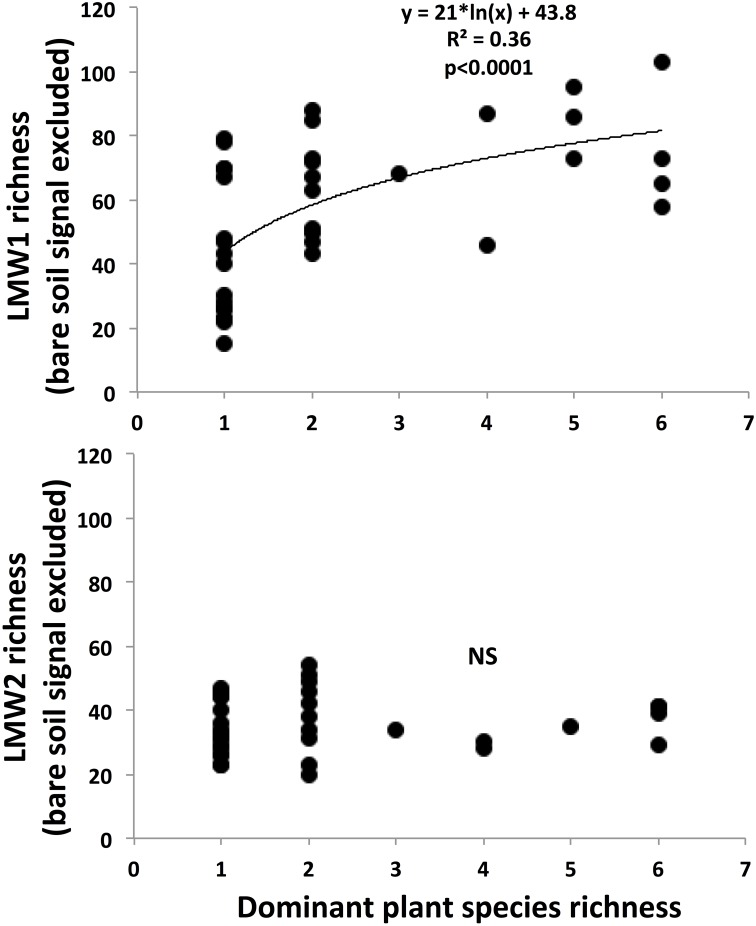
Effect of realized plant species richness on the richness of soil LMW1 compounds (top) and soil LMW2 compounds (bottom). Individual peaks corresponding to the bare soil background were excluded. Data for the richness of LMW1 organic compounds were fitted with a logarithmic function. NS: no significant linear or logarithmic relationship.

A positive, logarithmic relationship was observed between LMW1 compound richness and plant species richness (R^2^ = 0.36, p<0.001) (Fig 3a). Mean LMW1 richness, bare soil background excluded, increased from around 40 compounds for plant monocultures up to around 80 compounds for mixtures with 6 plant species representing each >5% of total plant biomass. On the contrary, plant species diversity did not affect the LMW2 compound richness (Fig 3b). Mean LMW2 richness, bare soil background excluded, was around 35 whatever the plant diversity.

**Fig 4:**
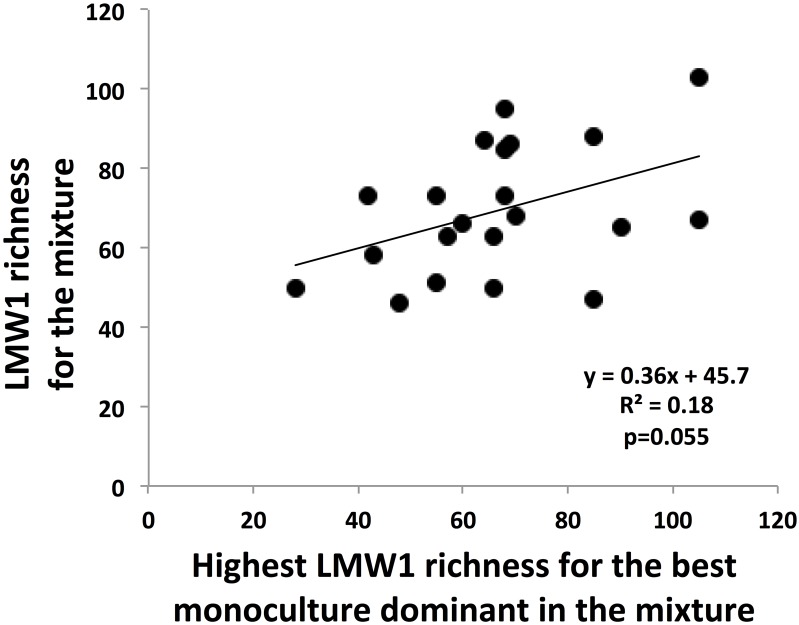
Relationship between the richness of soil LMW1 compounds observed for plant mixtures and the highest LMW1 compound richness observed for plant species present in the mixture when grown as monocultures. Individual peaks corresponding to the bare soil background were excluded. The line corresponds to the fitted linear regression.

In order to test a possible selection effect, the LMW1 compound richness measured for each plant mixture was compared to the maximum richness obtained among individual plant species present in the mixture. However, the observed correlation was weak and only marginally significant (p = 0.055; R^2^ = 0.18) (Fig 4). Furthermore, LMW1 richness for at least 38% of the plant mixtures was greater than the higher LMW1 richness observed among monocultures of plant species present in the mixture (Fig 4).

### Comparison of observed LMW1 richness with that computed assuming a perfect complementarity effect among plant species

**Fig 5:**
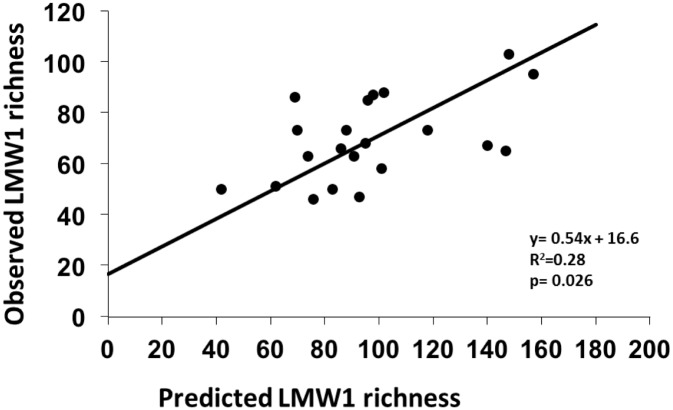
Role of complementarity effect within plant mixtures for the richness of soil LMW1 compounds. The richness of soil LMW1 compounds observed for plant mixtures is related to the LMW1 compound richness predicted for the same mixtures accounting for the LMW1 compounds profiles of the plant species present in each mixture and assuming a perfect complementarity effect (see S1 Fig). Individual peaks corresponding to the bare soil background were excluded. The line corresponds to the fitted linear functional regression.

When assuming a perfect complementarity effect among plant species (S1 Fig), a significant linear relationship was obtained between the predicted and observed LMW1 richness for plant mixtures (R^2^ = 0.28, p = 0.026) (Fig 5).

## Discussion

In the present work, the relationship between soil organic compound diversity and plant diversity was explored by applying up-to-date chromatographic profiling to characterize the diversity of extractible soil organic compounds for experimental grassland plots with contrasted plant species richness. This places our study at the crossroad of biochemistry, soil science and general ecology, which is the case for few studies [34–35] and even more rarely for studies based on field trials inspired by state-of-the-art ecological concepts.

Our study focused on soil organic acids and phenolic compounds. Whereas sugars are known to be easily-used sources of carbon for most bacteria, they mainly originate (up to 80%) from microorganisms in soil [36], thus limiting the impact of plant inputs on these carbon sources. Bacteria also use organic acids as carbon sources and organic acids like succinate act as as carbon catabolite repressors for some bacterial taxa [37–38]. It has also been shown that these sources of carbon are more important than glucose for driving the level of soil processes such as N_2_O reduction [39]. Phenolics are also important compounds in the context of plant-soil-microorganisms interactions. Microorganisms degrading phenolic compounds are diverse and widespread in soil, especially in the rhizosphere, and some taxa use phenolics as main organic carbon sources rather than other carbon sources [40]. For some strains, aromatic compounds are preferred carbon sources over glucose [41–42], and their degradation pathways are not repressed when organic acids are also present [41]. In addition, beyond sole trophic interactions, phenolic compounds can also impact key soil processes and microbial functional groups like soil denitrification and denitrifiers [43–44].

### Plant effect on soil organic compound richness: No effect for phenolic compounds, strong effect for organic acids

Our results show that plant diversity has a contrasted effect on the richness of either LMW1 or LMW2 compounds. Several results reported here show that plant effect on the diversity of LMW2 compounds is weak: (1) the level of LMW2 richness observed for bare soil was similar to that observed for most soils with plants, (2) the range of values of LMW2 richness was small among plant species grown as monocultures, (3) plant functional group or family had no significant effect on LMW2 richness, and (4) plant species richness did not significantly influence LMW2 richness. This suggests that, even 4 years after the beginning of the experiment, plant effect was less important than the soil background effect determined by the type of soil and previous plant cover and management regime at the experimental site.

Several papers have reviewed the type of phenolic compounds produced by different plant species [45–47]. The quantity and type of phenolics differ among plant species, but published studies generally only consider plant tissue composition, and rarely their release and availability in the soil. Focusing on the amount and not the diversity of soil phenolics, Meier et al. [48] compared phenolics concentration in soils planted with different plant species (phenolics-rich or phenolics-poor species) and bare soil, and quantified the effect of plant cover removal. Four years after plant cover removal, the phenolics concentration in bare soils (previously planted with phenolics-rich or phenolics-poor species) tend to decrease but are rarely significantly different to planted soils (only 2 dates/11 and only for phenolics-rich species) [48]. This is also consistent with the slow build up of stable organic matter in soil observed after grassland establishment [49]. In our case, using only one sampling time (for practical constraints) could have restricted our ability to detect any plant effects on soil phenolic compound diversity. An alternative explanation for the lack of plant effect on the richness of soil phenolic compounds is that soil LMW phenolics are often considered to mostly originate from the leaching and degradation of litter (mostly the aboveground one, but also the belowground litter). In the case of the biodiversity Jena experiment, grasslands are managed as hay meadows mown two or three times a year without restitution of plant biomass. This management may strongly decrease the phenolic input coming from aboveground litter and could contribute to reduce plant effect on soil phenolic diversity. The richness and the composition of phenolic compounds extracted from soil could then be more related to the common, former soil history and grassland management, rather than recent plant inputs. Another explanation for this lack of plant effect could be similar to that given by Sauheitl et al. [35] who assumed that the absence of the plant diversity effect on the free amino acid pool in soil could result from a rapid degradation of these compounds in soil. Since phenolic compounds can be used as substrate by soil microorganisms [50] a rapid use of LMW phenolic compounds derived from plants can occur, which may dampen plant effects on the composition of these compounds in soil. Analysis of the fate and speed of degradation of these types of compounds using, e.g., ^13^C labelling and the SIMS methodology could be used to test this hypothesis. However, studies have shown that LMW phenolics originate in part from the synthesis by microorganisms [51]. This may contribute to the important role of the soil background observed for these compounds in this experiment, and may explain why the LMW2 compound richness was higher for the bare soil than for a few plant monocultures.

On contrary, several results reported here show that plant effect on the diversity of soil organic acids is strong: (1) the level of organic acid richness observed for bare soil was lower than that observed for all planted soils, (2) the range of values of soil organic acid richness was large among plant species grown as monocultures, (3) plant family had a marginally significant effect on soil organic acid richness, and (4) plant species richness significantly influenced soil organic acid richness. Whereas phenolic compounds, amino acids or sugars are derived from polymeric forms (plant residues) present into SOM so that their stocks can be renewed by the activity of extracellular enzymes on these polymeric forms [52], there is no such reservoir for organic acids in soil. Replenishment of the soil organic acids pool hence depends almost only on the release from plant cells, which could explain why the influence of plant cover and plant species diversity is higher on soil organic acids than on soil phenolics.

### Effects of plant identity, family and plant functional group on soil organic acid richness

Soil organic acid richness in planted soil was influenced by plant identity, with values ranging from 38 to 138 depending on plant species. This is consistent with previous studies showing that the amount and the composition of organic acids released by different plant species, mainly as root exudates, vary between species. However, most of these studies analyzed only a few specific organic acids, typically around ten compounds for a few particular plant species [53]. To our knowledge, no study has characterized the range of soil organic acids present in soil in relation to plant species.

Soil organic acid richness was influenced, though marginally, by plant family since soil underneath Apiaceae had a higher richness of these compounds than soil underneath Asteraceae, Fabaceae, Plantaginaceae and Poaceae. Apiaceae family includes several species used as condiments or as medicinal plants, properties that could be attributed to their specific phytochemistry [54]. However, phytochemical analyses for this plant family focused on the essential oils produced in the aerial parts [55] rather than the compounds produced and released into soil by these plants. In contrast, the plant functional groups *a priori* defined when the Jena Biodiversity Experiment was set up did not influence the diversity of soil organic acids. This is not surprising, because these functional groups have been defined according to plant morphological and phenological characteristics along with ability to assimilate atmospheric N_2_ [32], without any account for other characteristics, in particular plant biochemistry. This clearly shows that the definition of 'functional groups' can strongly differ according to the type of ecosystem characteristics and functions considered, and that groups defined a posteriori can substantially differ as compared to groups defined initially, as already reported by Wright et al. [56].

### Effects of plant species richness on the richness of soil organic acids: Importance of complementarity effect among plant species

We hypothesized that an increase of plant species richness would induce an increase of the richness of soil LMW organic compounds. This hypothesis is implicit in many published studies [25–26] but to our knowledge has never been demonstrated. It is based on the assumption that a more diverse plant community both more often includes at least one plant species producing a high number of compounds (equivalent to a 'selection effect') and involves a more diverse input of LMW organic compounds by the different plant species to soil (equivalent to a 'complementarity effect'). Depending on the type of the LMW compounds, such an increase of the diversity of soil organic compounds with increased plant species richness is however not always observed as shown for free amino acids [35] and LMW2 compounds (including many phenolics) (our study). Nevertheless, for LMW1 compounds (essentially organic acids), the richness of soil organic acids—bare soil background excluded—was nearly twofold higher for plots with 6 dominant plant species as compared to plots with only one. We analyzed the results beyond the simple observation of this pattern to understand to what extent a selection or complementarity effect explained the relationship.

Selection effect did not have the major role here. Indeed, the relationship was weak between the richness of soil LMW1 observed for plant mixtures and the highest LMW1 richness observed for plant species present in the mixtures when grown as monocultures (Fig 4); and more importantly, in around 40% of the studied plant mixtures, LMW1 richness for mixtures was higher than the highest value observed for relevant plant monocultures. This is equivalent to a strong transgressive overyielding effect, as defined by Trenbath (1974) [57], since the rate of occurrence of transgressive overyielding in the literature is generally quite low (e.g. 12% and 23% when considering primary productivity according to the metanalysis of Cardinale et al. [58]). In contrast, a clear complementarity effect among plant species was observed (Fig 5): when different plant species were associated with contrasted spectra of soil LMW1, mixtures of these plant species showed particularly high richness of these soil compounds. In contrast, when different plant species were associated with more similar spectra of soil LMW1, mixtures of these plant species showed relatively low richness of these soil compounds. This explains that the richness of LMW1 varied more than twofold among the plant mixtures studied. This complementarity effect seems not to be simply additive since the observed richness of soil LMW1 richness for plant mixture were often lower than the richness expected from values observed for plant monocultures. However, this is particularly true for high values of LMW1 richness (Fig 5). This could be due to a methodological limitation, the number of peaks detected with the chromatographic approaches used tending to saturate for soil samples having the highest values of compounds richness. Another explanation is that plant biochemistry and compounds input into soil differed in monocultures or in mixtures due to interactions between plants, and that interspecific competition tended to decrease the mean number of LMW1 produced and brought into soil by a given plant species. However, our results clearly demonstrate that plant species richness strongly affects the richness of LMW1 compounds mainly through a complementarity effect among plant species.

## Conclusion

Our study provides new insights to the analysis of biodiversity effect on ecosystem characteristics. By assessing the effect of plant diversity manipulated *in situ* on the diversity of extractible soil organic compounds, this study connects plant and general ecology with biochemistry and soil science, accounting for a broad range of compounds and a high number of plant species and plant mixtures rather than focusing on particular compounds for a few plant species. This is needed to further understand the mechanisms underlying the effect of plant diversity on ecosystem processes, particularly belowground characteristics and functions that are less studied [59].

A major result of this study is that plant identity influences the diversity of LMW soil organic compounds and that plant species richness increases the richness of soil organic compounds, at least for LMW1 compounds. Since LMW organic acids are important substrates for soil microorganisms and since the preferential use of different compounds differs between soil microbial taxa [22], this could explain the influence of plant identity and diversity on soil microbial community structures and activities (as reported in many studies [60–62]) and ultimately soil functioning.

## Supporting information

S1 Fig Method used to predict the LMW richness in soil harboring a mixture of plants. (DOC)

S1 Table List of the plant species studied, with the abbreviation used in Fig 1. (DOC)

S1 Text Optimized procedures for chromatographic analysis of phenolic compounds by HPLC. (DOC)
