# Supplementary material for: Effect of plant diversity on the diversity of soil organic compounds
Source: PLoS One. 2017 Feb 6;12(2):e0170494. doi: 10.1371/journal.pone.0170494 (PMC5293253; doi:10.1371/journal.pone.0170494)
Supplement: S1 Fig — (DOC) [file pone.0170494.s001.doc]

**Supporting information:**

**Effect of Plant Diversity on the Diversity of Soil Organic Compounds**

**Lamiae El Moujahid ^1^, Le Roux Xavier ^1,*^, Serge Michalet ^1,2^, Florian Bellvert ^1,2^, Alexandra Weigelt ^3,4^ & Franck Poly ^1^**

**S1 Figure. Method used to predict the LMW compound richness in soil harboring a mixture of plants** by assuming a sole role of complementarity effect among plant species. The method is schematized for the case of a 2-plant-mixture. Given the LMW compound profiles observed for plants A and B growing separately as monocultures (upper panels), the profile for the soil where these plants grow in mixture is predicted by summing all the detected peaks (lower panel). Each bar represents the presence of an LMW compound. In the lower panel, grey bars correspond to peaks detected for both monocultures, whereas white or black bars correspond to peaks detected only for monoculture of plants A or B, respectively.
