# Supplementary material for: Effect of plant diversity on the diversity of soil organic compounds
Source: PLoS One. 2017 Feb 6;12(2):e0170494. doi: 10.1371/journal.pone.0170494 (PMC5293253; doi:10.1371/journal.pone.0170494)
Supplement: S1 Table — (DOC) [file pone.0170494.s002.doc]

**Supporting information:**

**Effect of Plant Diversity on the Diversity of Soil Organic Compounds**

**Lamiae El Moujahid ^1^, Le Roux Xavier ^1,*^, Serge Michalet ^1,2^, Florian Bellvert ^1,2^, Alexandra Weigelt ^3,4^ & Franck Poly ^1^**

**S1 Table.** **List of the plant species studied**, with the abbreviation used in Fig 1 and values of LMW1 and LMW2 compound richness detected in soil under each monoculture.

| **Abbreviation** | **Full name** | **LMW1 richness** | **LMW2 richness** |
| --- | --- | --- | --- |
| Ach mil | *Achillea millefolium* | 69 | 61 |
| Alo pra | *Alopecurus pratensis* | 64 | 67 |
| Ant odo | *Anthoxanthum odoratum* | 107 | 66 |
| Ant syl | *Anthriscus sylvestris* | 106 | 82 |
| Arr ela | *Arrhenatherum elatius* | 92 | 68 |
| Ave pub | *Avenula pubescens* | 84 | 89 |
| Bro ere | *Bromus erectus* | 82 | 67 |
| Bro hor | *Bromus hordeaceus* | 60 | 73 |
| Car car | *Carum carvi* | 98 | 51 |
| Car pra | *Cardamine pratensis* | 74 | 57 |
| Cir ole | *Cirsium oleracerum* | 59 | 63 |
| Cyn cri | *Cynosurus cristatus* | 96 | 75 |
| Dac glo | *Dactylis glomerata* | 82 | 59 |
| Dau car | *Daucus carota* | 99 | 65 |
| Fes pra | *Festuca pratensis* | 52 | 57 |
| Fes rub | *Festuca rubra* | 73 | 82 |
| Gal mol | *Galium mollugo* | 59 | 89 |
| Ger pra | *Geranium pratense* | 75 | 63 |
| Gle hed | *Glechoma hederacea* | 71 | 59 |
| Her sph | *Heracleum sphondylium* | 86 | 62 |
| Hol lan | *Holcus lanatus* | 75 | 70 |
| Kna arv | *Knautia arvensis* | 81 | 34 |
| Lat pra | *Lathyrus pratensis* | 54 | 57 |
| Leo aut | *Leotodon autumnalis* | 66 | 74 |
| Leo his | *Leotodon hispidus* | 93 | 65 |
| Leu vul | *Leucanthemum vulgare* | 86 | 78 |
| Lot cor | *Lotus corniculatus* | 138 | 68 |
| Med lup | *Medicago lupulina* | 85 | 60 |
| Med var | *Medicago varia* | 60 | 88 |
| Ono vic | *Onobrychis viciifolia* | 47 | 71 |
| Pas sat | *Pastinaca sativa* | 91 | 68 |
| Phl pra | *Phleum pratense* | 54 | 38 |
| Pim maj | *Pimpinella major* | 124 | 65 |
| Pla lan | *Plantago lanceolata* | 58 | 87 |
| Pla med | *Plantago media* | 90 | 63 |
| Poa pra | *Poa pratensis* | 59 | 59 |
| Poa tri | *Poa trivialis* | 61 | 41 |
| Pri ver | *Primula veris* | 87 | 74 |
| Pru vul | *Prunella vulgaris* | 57 | 71 |
| Ran acr | *Ranunculus acris* | 44 | 57 |
| Ran rep | *Ranunculus repens* | 68 | 39 |
| Rum ace | *Rumex acetosa* | 99 | 65 |
| San off | *Sanguisorba officinalis* | 63 | 72 |
| Tar off | *Taraxacum officinale* | 90 | 50 |
| Tra pra | *Tragopogon pratensis* | 65 | 82 |
| Tri cam | *Trifolium campestre* | 61 | 80 |
| Tri dub | *Trifolium dubium* | 56 | 76 |
| Tri fla | *Trisetum flavescens* | 120 | 62 |
| Tri hyb | *Trifolium hybridum* | 71 | 68 |
| Tri pra | *Trifolium pratense* | 76 | 46 |
| Tri rep | *Trifolium repens* | 98 | 54 |
| Ver cha | *Veronica chamaedrys* | 82 | 72 |
| Vic cra | *Vicia cracca* | 51 | 55 |
