# Supplementary material for: Effect of plant diversity on the diversity of soil organic compounds
Source: PLoS One. 2017 Feb 6;12(2):e0170494. doi: 10.1371/journal.pone.0170494 (PMC5293253; doi:10.1371/journal.pone.0170494)
Supplement: S1 Text — (DOC) [file pone.0170494.s003.doc]

**Supporting information:**

**Effect of Plant Diversity on the Diversity of Soil Organic Compounds**

**Lamiae El Moujahid ^1^, Le Roux Xavier ^1,*^, Serge Michalet ^1,2^, Florian Bellvert ^1,2^, Alexandra Weigelt ^3,4^ & Franck Poly ^1^**

**S1 Text.** **Optimized procedures for chromatographic analysis of phenolic compounds by HPLC.**

For polar extracts, gradient was established using solvents A (formic acid 0.4% in distilled water), and B (formic acid 0.4% in methanol). A gradient elution combining solvent A and B was carried out at a flow rate of 1 ml/min, following the proportions : 0% solvent B from 0 to 10 min, 0 to 100% B from 10 to 40 min, 100% B from 40 to 46 min, 100 to 0% B from 46 to 50 min, 0% B from 50 to 58 min.

For apolar extracts, gradient was established using solvents A (formic acid 0.4% in distilled water), and B (formic acid 0.4% in acetonitrile). A gradient elution combining solvent A and B was carried out at a flow rate of 0.8 ml/min: 0% solvent B from 0 to 4 min, 0 to 7% B from 4 to 20 min, 7 to 27% B from 20 to 35 min, 27 to 100% B from 35 to 40 min, 100% B from 40 to 45 min, 100 to 0% B from 45 to 48 min, 100% B from 48 to 55 min.
